# Psychosocial care for patients included in a mental health specialised service for migrant population: a demographic study

**DOI:** 10.1192/j.eurpsy.2026.10987

**Published:** 2026-07-31

**Authors:** A. Ferro, P. Cristóbal-Narváez, M. Alfonso, H. Sainz-Elias, J. M. Haro, Y. Osorio

**Affiliations:** 1 Parc Sanitari Sant Joan de Déu, Sant Boi, Spain; 2 Research Department; 3 Servicio de Atención a la Migración en Salud Mental (SATMI), Parc Sanitari Sant Joan de Déu, Sant Boi, Spain

## Abstract

**Introduction:**

Migrant population presents specific mental health care needs, as they face a higher risk of psychological distress. Challenges are presented, including language barriers, socioeconomic conditions, and legal status. Tailored mental health services are crucial for effectively addressing these needs. However, in Spain, there is limited evidence on the functioning and outcomes of such services. The *Servei d’Atenció a la Salut Mental de la Població Migrada* (SATMI) has been providing specialised mental health care for migrant population in Barcelona since 1997.

**Objectives:**

This study aims to describe the sociodemographic and clinical profiles of migrants receiving care through SATMI program. Thus, to analyse their service trajectories in order to inform future improvements in mental health care provision.

**Methods:**

This retrospective descriptive study analyses sociodemographic, clinical, and service-use data collected from all individuals who attended SATMI during 2023. Variables include age, sex, nationality, country of birth, language, and need for an interpreter. At the same time, social and clinical data are also included, such as migration-related categories (refugee, unaccompanied minor, homeless, or family reunification), residence permit status, psychiatric diagnosis, and clinical course (including the number and type of sessions). Descriptive statistical analysis is applied to examine participant profiles and attendance patterns.

**Results:**

Preliminary findings indicate that 262 individuals accessed SATMI services in 2023. The majority were men (146; 55,7%). Regarding migration categories, 47 participants were identified as refugees, 18 as unaccompanied minors, and 17 as homeless. Following DSM – V main diagnose criteria, 14 categories were identified. Among them, the most common were (i) trauma and stressor - related disorders (96; 36,6%), (ii) psychotic disorders (40, 15,3%), (iii) depressive disorders (38, 14,5%), and (iv) anxiety disorders (20; 7,6%). Data highlights the diversity of needs addressed within the program.

**Conclusions:**

This study provides data on specialised migrant mental health services in Spain. The results show the complexity of the attended population. This supports the need for specialized multidisciplinary teams. Limited availability of clinical information in this field highlights the importance of implementing systematic data collection for ongoing monitoring of services impact. In a nutshell, such demographic studies support a broader understanding of migrant mental health care needs.

**Disclosure of Interest:**

None Declared

